# Cell lineage tracing: Methods, applications, and challenges

**DOI:** 10.1002/qub2.70006

**Published:** 2025-05-08

**Authors:** Shanjun Mao, Chenyang Zhang, Runjiu Chen, Shan Tang, Xiaodan Fan, Jie Hu

**Affiliations:** ^1^ Department of Statistics Hunan University Changsha China; ^2^ Department of Statistics The Chinese University of Hong Kong Hong Kong China; ^3^ School of Mathematical Science Xiamen University Xiamen China

**Keywords:** cell lineage tracing, CRISPR‐Cas9, RNA velocity, single‐cell sequencing

## Abstract

Cell lineage tracing is a crucial technique for understanding cell fate and lineage relationships, with wide applications in developmental biology, tissue regeneration, and disease progression studies. Over the years, experimental cell lineage tracing methods have advanced from early labeling techniques to modern genetic tools such as CRISPR‐Cas9‐based barcoding, whereas computational methods have emerged to analyze high‐dimensional data from single‐cell sequencing and other omics technologies. This paper reviews both experimental and computational methods, highlighting their respective strengths, limitations, and synergies. Experimental techniques focus on labeling and tracking cells, whereas computational approaches reconstruct lineage relationships and model cellular dynamics. Despite significant progress, challenges remain, including issues with accuracy, resolution, multi‐omics integration, and scalability. Future directions involve improvements in experimental techniques and the development of computational methods enhanced by advancements in artificial intelligence. These innovations are expected to drive the field forward, offering potential applications in uncovering the mysteries of life.

## 1 INTRODUCTION

Cell lineage tracing is a pivotal technique in biological sciences that enables the mapping of cell development from progenitor cells to their differentiated descendants [1]. By elucidating the trajectories of individual cells and their progeny, cell lineage tracing provides profound insights into developmental processes, tissue regeneration, and disease progression [2, 3, 4]. The ability to track cells over time has not only revolutionized laboratory research but also had far‐reaching implications for medical practice and biotechnology.

Lineage tracing was first introduced in the laboratory and has since become indispensable for dissecting the complexities of developmental biology and stem cell dynamics [5, 6, 7, 8, 9]. It has enabled researchers to identify stem cell niches, understand differentiation pathways, and unravel the hierarchical organization of tissues. For instance, lineage tracing studies were crucial in mapping the development of the nervous system, hematopoietic lineages, and organogenesis in model organisms [4, 10, 11]. These insights have provided a foundation for manipulating cell fates in vitro and in vivo, with implications for regenerative medicine and tissue engineering [3, 12, 13].

Beyond the laboratory, the impact of cell lineage tracing extends to real‐world applications that affect human health and disease management. In oncology, tracing the lineage of cancer cells has illuminated the origins of tumor heterogeneity, mechanisms of metastasis, and the evolution of drug resistance [14, 15, 16]. This knowledge is instrumental in developing targeted therapies and personalized treatment strategies. In regenerative medicine, understanding cell lineage relationships informs the development of stem cell therapies aimed at repairing damaged tissues and organs [17]. Furthermore, lineage tracing contributes to developmental biology, enhancing the comprehension of congenital disorders and potential interventions [18].

The development of cell lineage tracing methods has evolved significantly over the past century. Early efforts in the field relied on observational studies and rudimentary labeling techniques, such as vital dyes, to follow cell movements in developing embryos [2, 19, 20]. These initial methods were limited by their lack of specificity and transient labeling. The advent of genetic labeling techniques marked a transformative period, introducing tools such as reporter genes and recombinase systems (e.g., Cre‐loxP) that allowed for more precise and heritable marking of cells [21, 22]. In parallel, advances in microscopy and imaging technologies enhanced the ability to visualize cells in vivo with greater resolution and over extended periods [23, 24].

With the growing volume and complexity of bio‐logical data, experimental methods alone are insufficient to meet the advanced requirements of lineage tracing. Consequently, computational methods have become essential tools for analyzing and interpreting lineage tracing experiments [25]. High‐throughput sequencing technologies and single‐cell analyses generate vast datasets that require sophisticated computational approaches to reconstruct lineage relationships accurately [26]. These methods complement experimental techniques, offering new avenues for modeling developmental processes and predicting cellular behaviors.

In this review, we will explore the current landscape of cell lineage tracing methodologies, categorizing them into two primary groups: experimental methods (wet lab) and computational methods (dry lab). The experimental methods section will discuss traditional and modern techniques used to label and track cells in biological systems. The computational methods section will delve into algorithms and models employed to analyze lineage data and reconstruct developmental trajectories. Experimental methods lay the foundation for computational approaches, whereas advances in computational techniques drive progress in experimental methodologies. Together, they form an essential synergy in cell lineage tracing. This interplay enables more precise, scalable analysis of lineage data, enhancing the ability to model complex biological systems. These innovations are poised to propel the field forward, transforming the understanding of cellular processes and unlocking applications with profound implications for human health and medicine.

## 2 OVERVIEW

### 2.1 Definition

**FIGURE 1:**
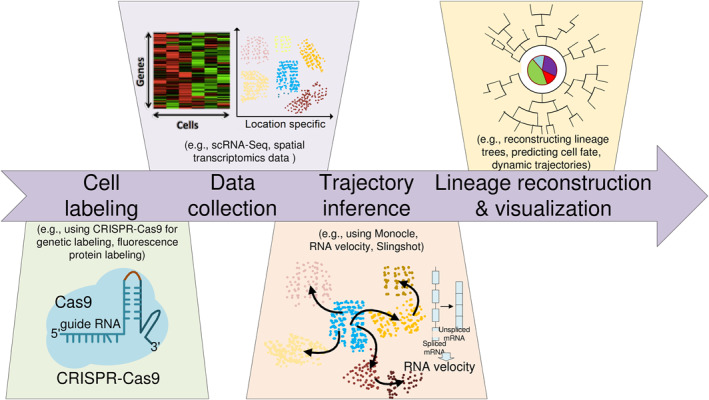
Typical workflow of computational cell lineage tracing. The workflow includes cell labeling, data acquisition, trajectory inference, and lineage reconstruction and visualization. First, target cells are labeled using cell labeling techniques (such as CRISPR‐Cas9 gene tagging or fluorescent protein labeling) to enable precise tracking of their evolutionary process in subsequent experiments. Next, scRNA‐seq or spatial transcriptomics is employed to collect transcriptional data from individual cells, providing gene expression information and spatial localization of each cell. Subsequently, trajectory inference tools, such as RNA velocity, Monocle, and Slingshot, are used to analyze the dynamic states and developmental trajectories of the cells, revealing potential changes in cell fate. Finally, lineage reconstruction algorithms are applied to build the cell developmental lineage, predict cell fate, and visualize the dynamic trajectories of cells throughout their development. This workflow provides a comprehensive framework for studying cell development, differentiation, and fate determination.

Lineage tracing is widely regarded as the gold standard for inferring the relationships between progenitor cells and their progeny. By labeling progenitor cells and examining their spatial distribution and marker expression at later time points, researchers can investigate lineage segregation events that occurred during the intervening period. Additionally, continuous labeling of progenitor cells at different stages allows for the construction of more detailed lineage trees and spectrum structures [1, 2, 27, 28]. Tracing all descendants of a single cell, known as prospective tracing, has a long history dating back to the earliest days of developmental biology. As technology has advanced—from molecular dyes to CRISPR barcoding—lineage tracing has evolved toward both finer resolution (distinguishing specific cells rather than populations) and broader applicability (tracking thousands of cells rather than a few) [29, 30]. The advent of high‐throughput sequencing technologies, such as single‐cell RNA sequencing (scRNA‐seq), has transformed traditional lineage tracing into single‐cell lineage tracing (SCLT). SCLT enables the simultaneous identification of ancestor‐descendant relationships through evolution of barcodes and the characterization of each cell’s molecular state using scRNA‐seq [31]. This integrated approach provides a comprehensive view of lineage relationships and molecular trajectories. Furthermore, thousands of clones can be analyzed in parallel to optimize lineage connectivity, enabling an unbiased and large‐scale investigation of cellular transitions [10, 32]. Figure 1 illustrates the typical workflow of computational cell lineage tracing, spanning from experiment to computation, including cell labeling, data acquisition, trajectory inference, lineage reconstruction, and visualization.

### 2.2 Benchmark datasets

Datasets underlying cell lineage tracing span a wide array of organisms and technologies, forming a foundational resource for developmental biology [33]. A canonical example is the extensively validated embryonic lineage of *Caenorhabditis elegans* (*C. elegans*), first established through meticulous microscopy by Sulston and colleagues [6]. This dataset provides a fully resolved, single‐cell roadmap from fertilization to the adult organism and has served as a gold standard reference for understanding cellular differentiation paths. More recently, the emergence of genomic technologies has enabled lineage reconstruction at unprecedented scales. For example, CRISPR‐based lineage barcoding combined with scRNA‐seq has facilitated large‐scale tracing of zebrafish development, capturing lineage trajectories across larvae and adult tissues [34]. Similarly, in mouse embryos, methods such as scGESTALT integrate CRISPR‐induced sequence variation with scRNA‐seq data to delineate complex lineage hierarchies [35]. Beyond classical model organisms, human cell studies have employed homing CRISPR systems to reconstruct developmental lineages in organoids and primary tissues [36]. Collectively, these datasets, ranging from the classical *C. elegans* lineage map to high‐throughput CRISPR‐driven reconstructions in vertebrates, constitute a robust framework for elucidating the branching points of cellular decision‐making and for informing regenerative medicine and disease modeling. Meanwhile, Table 1 presents available datasets that we have collected and organized, which may be valuable for future research.

**TABLE 1:** Benchmark datasets.

Dataset name	Species	Data type	Description	Primary use
*C. elegans* embryonic lineage [6, 37]	*C. elegans*	Transcriptomics (scRNA‐seq)	The embryonic cell lineage of *C. elegans* from zygote to newly hatched larva.	Studying cell fate, gene expression dynamics, and lineage tracing.
Zebrafish development & lineage tracing [34, 38, 39, 40]	*Danio rerio*	Transcriptomics (scRNA‐seq)	Lineage tracing and scRNA‐seq of over 90,000 zebrafish cells.	Tracking cell lineage and differentiation during zebrafish development.
CARLIN mouse line [41]	*Mus musculus*	Genomics (CRISPR‐based, sequencing)	CRISPR‐based lineage tracing in a genetically defined mouse line.	Studying hematopoietic stem cell proliferation and transcriptional signatures.
MmuPV1‐harboring cells line [42]	*Mus musculus*	Genomics (reporter‐based)	Lineage tracing of MmuPV1‐harboring cells and their progeny.	Investigating papillomavirus‐host interactions and cell proliferation.
Embryonic endoderm fate study [35, 43]	*Mus musculus*	Genomics (HRP‐based)	Fate tracing of axial endoderm cells during early mouse embryonic development.	Studying embryonic endoderm development and lineage.
Human organoid development [36]	*Homo sapiens*	Transcriptomics (scRNA‐seq), genomics (CRISPR‐Cas9)	Lineage tracing in human organoids using CRISPR‐based systems.	Studying human organoid development.
mCherry‐tagged OE19 (esophageal cancer) [44]	*Homo sapiens*	Genomics (fluorescent labeling)	Lineage tracing in OE19 esophageal adenocarcinoma cells with mCherry marker.	Studying selection and lineage in esophageal cancer.
AML [45]	*Homo sapiens*	Transcriptomics (exome, RNA‐seq)	Lineage tracing of AML cells to study chemoresistance and DNMTi therapy efficacy.	Investigating AML chemoresistance and evaluating DNMTi treatment.
iPSC‐derived midbrain dopaminergic model [46]	*Homo sapiens*	Transcriptomics (scRNA‐seq), genomics (CRISPR‐Cas9)	Tracing dopaminergic progenitors from iPSCs for Parkinson’s disease modeling.	Modeling Parkinson’s disease and studying dopaminergic progenitor toxicity.

*Note*: This table summarizes cell lineage tracing datasets across various species and technologies, with applications ranging from developmental biology to disease modeling.

### 2.3 Evaluation metrics

When developing or introducing a new cell lineage tracing computational method, the following key indicators are used to evaluate its performance. Lineage tree accuracy, which assesses the precision of reconstructed lineage topologies compared to ground truth, remains a major measure [4]. Branching fidelity is another essential metric, quantifying the ability to capture bifurcations and divergence points in cellular development [47]. Temporal resolution is increasingly emphasized, particularly in studies integrating RNA velocity, as it measures the capability to infer dynamic cellular changes over time [48]. These metrics collectively ensure that lineage tracing methods provide robust and biologically meaningful insights.

## 3 CELL LINEAGE TRACING METHODS

Cell lineage tracing combines experimental and computational methods to map developmental trajectories and cell differentiation. Experimentally, lineage tracing involves labeling individual cells and tracking their progeny over time. One approach utilizes the CRISPR‐Cas9 system to introduce unique genetic barcodes into the genomes of individual cells. As cells divide, these barcodes accumulate mutations, allowing researchers to reconstruct lineage relationships by sequencing the barcodes in descendant cells [49, 50]. Another method involves genetically engineering cells to express fluorescent proteins, enabling visualization and tracking of cell divisions and migrations in living organisms. Additionally, retroviruses can be used to insert marker genes into the genomes of target cells, facilitating the tracking of these cells and their progeny over time [51].

Although experimental methods of cell lineage tracing are well established and provide important tools for studying cell fate, there are still many challenges. These include the massive data generated by the widespread use of single‐cell technologies, the time‐consuming and expensive nature of experimental methods, deficiencies in resolution and accuracy, as well as the high demands of dynamic trajectory analysis and the complexity of new biological hypotheses. Therefore, computational methods are necessary and indispensable. With experimental data, computational analyses can be employed to interpret the data and reconstruct cell lineage relationships. Both trajectory inference algorithms and phylogenetic reconstruction methods rely on the experimental data mentioned above [47, 52, 53, 54].

**FIGURE 2:**
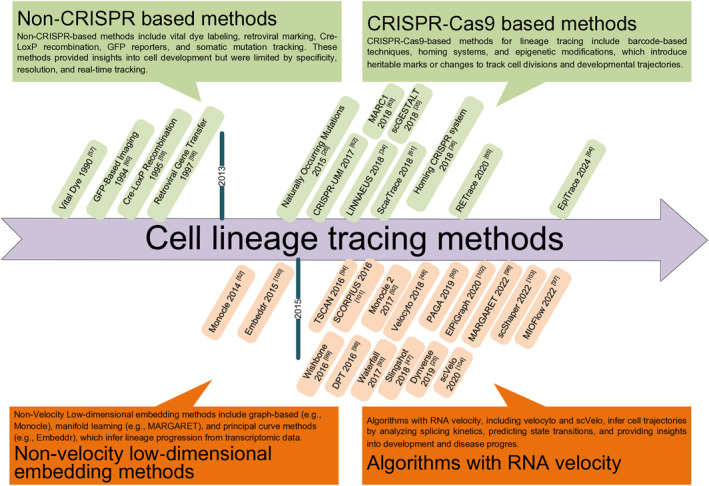
The timeline of methods related to cell lineage tracing. The experimental methods are categorized based on the use of CRISPR‐Cas9, whereas the computational methods are classified according to whether they rely on RNA velocity.

The experimental and computational methods related to cell lineage tracing are not mutually exclusive but complement each other. The experimental techniques generate detailed real lineage data, although computational analysis interprets these data to reconstruct the developmental pathways. The former serves as the basis for the latter, and the latter relies on the former to deepen understanding and expand knowledge, with both providing insights into the mechanisms of cell lineage tracing. Figure 2 presents the timeline of methods related to cell lineage tracing.

### 3.1 Experimental methods

Experimental methods for cell lineage tracing involve directly manipulating cells and biological systems in the laboratory to track lineage using molecular or physical markers [1, 55]. These techniques allow researchers to label individual cells and monitor their progeny over time, offering valuable insights into developmental processes and cellular differentiation. Among these methods, CRISPR‐Cas9‐based approaches have emerged as a powerful tool for lineage tracing by introducing heritable genetic barcodes or scars into the genome [56]. These barcodes accumulate over successive cell divisions, enabling precise reconstruction of lineage trees and cellular trajectories [12]. Based on whether CRISPR‐Cas9 is employed, experimental methods can be categorized into two distinct groups. Table 2 summarizes these methods in detail, along with the types of experimental data and related descriptions.

**TABLE 2:** Experimental methods.

	Method	Method description	Data description
Non‐CRISPR based	Vital dye [57]	Track neural crest cell migration using vital dyes.	Zebrafish embryos, transient non‐specific dyes.
	Retroviral gene transfer [58]	Long‐term lineage tracing using viral vectors.	Hematopoietic stem cells labeled by retroviral vectors.
	Cre‐LoxP recombination [59]	Tracing neural lineage in transgenic mice.	Cell‐type‐specific recombination for lineage tracing.
	GFP‐based imaging [60]	Real‐time visualization of cellular migration.	GFP markers used for dynamic imaging.
	Naturally occurring mutations [26]	Reconstruct lineage trees from genetic alterations.	Retrospective analysis of microsatellite repeats and epigenetic changes.
CRISPR‐Cas9 based	LINNAEUS [34]	Lineage tracing with CRISPR‐induced indels.	Zebrafish larvae and adult tissues (heart, liver, pancreas, telencephalon).
	ScarTrace [61]	CRISPR scars for lineage tracing.	Zebrafish embryos and cell lineage patterns.
	CRISPR‐UMI [62]	Lineage mapping with CRISPR and UMIs.	Functional genomic screens across mammalian cells.
	scGESTALT [35]	Large‐scale lineage tracing using RNA‐seq and CRISPR.	Mouse embryo and zebrafish lineage with single‐cell transcriptomes and scars.
	Homing CRISPR system [36]	Continuous accumulation of heritable edits for lineage tracing.	Developmental lineage in mouse tissues and organoids.
	MARC1 [63]	CRISPR barcoding for long‐term lineage tracing.	Human and mouse cells, with a focus on organs (lung, liver).
	EpiTrace [64]	Simultaneous lineage and epigenetic tracking with CRISPR.	Tracking lineage and epigenetic modifications during development and diseases.
	RETrace [65]	Inference of evolutionary history from CRISPR barcodes.	Reconstruction of lineage trees in microbial and mammalian populations.

*Note*: These methods are divided into two categories based on whether CRISPR‐Cas9 technology was used, with different methods and their descriptions introduced in detail, along with the types of experimental data and related explanations.

#### 3.1.1 Non‐CRISPR‐based methods

Before the advent of CRISPR‐Cas9 technology, various methods were developed to trace cell lineages, each contributing valuable insights into cellular development and differentiation. Early techniques such as vital dye labeling marked cells with fluorescent dyes to observe their movements and divisions. These studies, exemplified by Ref. [57], provided foundational observations of developmental processes such as neural crest cell migration but were limited by transient marking and lack of specificity.

Advancements in molecular biology introduced retroviral‐mediated gene marking, which enabled researchers to label target cells with viral vectors carrying marker genes. This technique facilitated long‐term lineage tracing and was particularly effective in studying hematopoietic stem cells. For instance, Persons et al. used retroviral vectors to trace stem cell progeny, revealing detailed insights into blood cell development [58]. However, retroviral systems faced challenges such as random insertion sites and potential genomic perturbations.

Genetic recombination systems such as Cre‐LoxP further improved specificity in lineage tracing. These systems allowed for cell‐type‐specific and heritable genetic labeling by inducing recombination events at targeted genomic loci. Cre‐LoxP technology was instrumental in neuroscience, mapping neural circuits with precision and offering a model for studying cell fate decisions [59]. Despite their versatility, these methods often required sophisticated genetic engineering and a lack of temporal resolution.

The introduction of fluorescent protein reporters, such as GFP, marked another milestone in lineage tracing. These genetically encoded markers facilitated live‐cell imaging, enabling researchers to observe cellular behaviors dynamically in living organisms. Chalfie et al. showcased the use of GFP in real‐time tracking of cellular interactions, though the technique was constrained by its reliance on external fluorescence detection and potential phototoxicity [60].

Lastly, retrospective methods based on naturally occurring somatic mutations provided an alternative to prior labeling. These methods utilized genetic or epigenetic changes such as microsatellite repeats and chromatin modifications as intrinsic lineage markers. High‐throughput sequencing, as used by Ref. [26], greatly enhanced the ability to detect these subtle changes, reconstructing lineage trees without experimental perturbation. However, these methods often struggled with low resolution and lacked the capacity to track real‐time dynamics.

Despite their limitations, pre‐CRISPR methods laid the foundation for modern lineage tracing by addressing key biological questions and advancing the understanding of developmental processes. They provided the conceptual and technological basis for the transformative approaches enabled by CRISPR‐Cas9.

#### 3.1.2 CRISPR‐Cas9‐based methods

CRISPR‐Cas9, an adaptive immune system discovered in microbes, has transformed the field of genome engineering [66, 67, 68]. This versatile tool enables precise RNA‐guided genome editing, transcriptional regulation, and epigenetic modifications in eukaryotic cells [69]. Among its numerous applications, CRISPR‐Cas9 has been widely adopted for cell lineage tracing, providing groundbreaking insights into developmental biology and stem cell research.

At the core of CRISPR–Cas9 based cell lineage tracing methods lies the ability to introduce heritable genetic marks into the genome, which are stably passed down to progeny cells. These marks, either synthetic barcodes or natural scars (mutations), accumulate progressively as cells divide, serving as unique molecular records of lineage relationships [70]. By sequencing these edits at the experiment’s endpoint or throughout its progression, researchers can reconstruct lineage trees and developmental trajectories [71]. Moreover, integrating these methods with scRNA‐seq or other omics technologies allows lineage information to be linked with cell states or functional data, offering high‐resolution and scalable insights into cellular development and hierarchy [72, 73]. To date, there are many practices in this field. According to the mechanism of lineage recording, these methods can be broadly categorized into barcode‐based methods, homing CRISPR systems and epigenetic methods.

Barcode‐based methods rely on creating unique, heritable genetic identifiers that are passed down to progeny cells. These identifiers, often in the form of synthetic DNA or RNA sequences, are modified as cells divide to record lineage relationships [74, 75]. LINNAEUS, a notable method, uses genome editing of transgenic reporter genes to generate lineage barcodes, enabling simultaneous lineage tracing and transcriptome profiling in zebrafish [34]. ScarTrace, a technique using CRISPR‐Cas9‐induced genetic scars in zebrafish, enables massively parallel whole‐organism lineage tracing, revealing the complex relationship between embryonic progenitors and adult cell types [61, 76]. Not only focused on CRISPR‐Cas9, CRISPR‐UMI enhances lineage tracing by incorporating unique sequence tags alongside CRISPR‐induced edits, enabling error correction and improved resolution in lineage reconstructions [62]. The scGESTALT method combines CRISPR‐Cas9 barcode editing with scRNA‐seq to generate lineage trees with hundreds of branches, providing insights into cell type relationships [35]. Liao et al. proposed the DNA Typewriter [77], which leverages prime editing to facilitate sequential insertions into an engineered genomic region, effectively capturing the temporal relationships among recorded events in mammalian cells. The DNA Typewriter can be applied effectively in two areas: recording sequential transfection events with programmed barcode insertions using prime editing, and recording lineage information during the expansion of a single cell into many cells. However, many clonal lineage identification methods using genetic barcodes or CRISPR‐Cas9 induce DNA damage, potentially altering the transcriptome and drug sensitivity. To address this, Wild et al. developed WILD‐Seq [78], which leverages expressed barcodes and population bottlenecking in syngeneic mouse models combined with scRNA‐seq. This approach ensures robust identification of clonal gene expression signatures and differential clonal abundance before and after therapy, enabling precise characterization of drug resistance mechanisms in vivo.

The homing CRISPR system utilizes homing guide RNAs (hgRNAs) that direct the CRISPR‐Cas9 nuclease to their own DNA loci, creating diverse mutational outcomes. This system allows for the generation of unique genetic barcodes that can be inherited by daughter cells, enabling the reconstruction of lineage trees [36, 79]. It also overcomes limitations of traditional Cre‐loxP systems by capturing the heterogeneous nature of complex organs such as the kidney [80]. The MARC1 mouse line, which carries multiple hgRNAs, has been used to create developmentally barcoded mice. These barcodes record lineage‐specific mutations that can be sequenced to reconstruct the earliest lineages and investigate developmental processes, such as brain axis development [4, 63]. Liu et al. developed a substitution mutation‐aided lineage‐tracing system (SMALT) [81]. This system utilizes a deaminase to target the DNA barcoding sequence via the functional domain of the iScel protein. The C base within the sequence is deaminated to a U base, and during DNA replication, the U base pairs with an A base, resulting in a C‐to‐T mutation. Compared to the traditional CRISPR‐Cas9 gene editing system, SMALT exhibits a faster mutation rate and provides higher resolution in cell lineage tracing, making it suitable for tracing lineages in complex multicellular biological tissues.

Epigenetic methods use nuclease‐dead Cas9 fused with epigenetic modifiers to introduce changes in DNA methylation or histone modifications, recording lineage without altering the genetic sequence [82]. In contrast to genetic barcoding methods, epigenetic lineage tracing has the advantage of being minimally invasive and not requiring genetic engineering. A novel approach called EpiTrace utilizes single‐cell chromatin accessibility sequencing to assess the fraction of opened clock‐like loci, which correlate with cell age. This method provides a means to trace lineages across various species and cell types, revealing insights into processes such as hematopoiesis and tumor biology [64]. Another method, RETrace, combines microsatellite lineage tracing with methylation profiling, allowing for simultaneous lineage and cell type characterization from single cells, and achieves high accuracy in lineage tracing by reducing amplification noise [65].

Although CRISPR‐Cas9 has dominated the field of gene editing, the development of improved technologies derived from the CRISPR/Cas system, including CRISPR/Cas12a, CRISPR/Cas13, base editing, and prime editing (PE), provides additional tools with different mechanisms that further expand their frontier applications in cell lineage tracing [83, 84, 85, 86, 87]. For example, PRIME‐Del [88], a method based on prime editing, uses a pair of plasmids to edit sgRNA to induce deletions. The targeted DNA strand of these sgRNAs is complementary, allowing for the precise determination of both the deletion site and the repair outcome. Compared to CRISPR‐Cas9 and sgRNA pairs, PRIME‐Del shows significantly higher precision when programming deletions up to 10 kb, with an editing efficiency of 1%–30%. PRIME‐Del can be widely used for precise and flexible genome deletion programming, epitope tagging, and potentially genome rearrangement programming. Similarly, bi‐directional prime editing (Bi‐PE) [89, 90] utilizes two PE guide RNAs, enabling broader and higher editing efficiency. This method has the potential to modify, delete, integrate, and replace larger genomic sequences while editing multiple bases simultaneously. The Bi‐PE strategy expands the editing scope of the prime editing system, enhancing both its efficiency and accuracy.

### 3.2 Computational methods

Computational methods for cell lineage tracing analyze biological data to infer lineage relationships and reconstruct developmental trajectories. A notable approach in this domain is RNA velocity, which estimates future gene expression states by analyzing the abundance of unspliced (nascent) and spliced (mature) mRNA transcripts [48]. This dynamic information provides insights into the direction and speed of cellular state transitions.

Several computational methods were developed for lineage tracing that do not rely on RNA velocity [47, 91]. These methods utilize various statistical and machine learning techniques to reconstruct developmental pathways and elucidate cellular differentiation processes. So computational methods can be roughly divided by whether they use the information of RNA velocity. Table 3 provides an overview of these methods and their key features.

**TABLE 3:** Computational methods.

	Method	Data description	Data/Resource	Coding language/Resource
Non‐velocity low‐dimensional embedding	Monocle [52]	Primary human myoblasts.	GSE52529	R
	Monocle [292]	Gene regulation and RNA processing in stimulated immune cells.	Gene expression omnibus	R
	Waterfall [93]	Genome‐wide dynamic expression profiles of TFs during adult neurogenesis.	GSE71485	R
	TSCAN [94]	Three datasets: (1) Human skeletal muscle myoblasts (HSMM). (2) scRNA‐seq samples collected after stimulating bone‐marrow‐derived dendritic cells by lipopolysaccharide. (3) Hippocampal quiescent neural stem cells.	TSCANdata	R
	Slingshot [47]	Three datasets: (1) HSMM. (2) Olfactory epithelium (OE) data. (3) Synthetic data.	Single cells, olfactory stem cell, splatter	R
	PAGA [95]	Adult planaria and the zebrafish embryo.	PAGA	Python
	MARGARET [96]	Three datasets: (1) Early human hematopoiesis datasets. (2) Embryoid body dataset. (3) scRNA‐seq data for colon differentiation.	Human cell atlas, embryoid body, GSE116222	Python
	MIOFlow [97]	Dynamic time course of human embryoid body differentiation over a period of 27 days.	MIOFlow	Python
	DPT [98]	Three datasets: (1) Early blood qPCR. (2) Mouse embryonic stem cells in DropSeq data. (3) Mouse myeloid progenitors MARS‐seq data.	GSE61470, GSE65525, GSE72857	Python & MATLAB
	Wishbone [99]	Two datasets: (1) Mouse thymus mass cytometry data. (2) Mouse myeloid scRNA‐Seq data.	Cytobank, GSE72857	Python
With RNA velocity	Embeddr [100]	Two datasets: (1) Differentiating human myoblasts. (2) Differentiating cells in the mouse distal lung epithelium.	Monocle, lung epithelium	R
	SCORPIUS [101]	10 scRNA‐seq datasets representing several types of dynamic processes: Cell differentiation, cell cycle and response upon external stimulus.	SCORPIUS	R
	ElPiGraph [102]	Hematopoietic data.	ElPiGraph	Python & R & MATLAB
	scShaper [103]	Imulators and the real data.	scShaper	R
	Velocyto [48]	scRNA‐seq with spliced and unspliced mRNA levels.	Available from published datasets	R & python
	Dynverse [25]	Integration of RNA velocity with trajectory inference methods.	Available from published datasets	R
	scVelo [104]	Integrates RNA velocity with gene expression dynamics.	Available from public repositories package	Python
	RNA velocity + lineage tracing [48]	Combines RNA velocity with CRISPR lineage tracing to study past and future cell states.	Published datasets custom analysis pipeline	Python

#### 3.2.1 Non‐velocity low‐dimensional embedding methods

Methods that do not incorporate RNA velocity rely solely on static transcriptomic snapshots, typically obtained through scRNA‐seq [105]. These approaches reconstruct lineage trajectories by analyzing transcriptional similarities and changes across cells [91, 106]. In the absence of kinetic information, such as that provided by RNA velocity, these methods often employ low‐dimensional embedding or dimension reduction to infer lineage progression in pseudotime. Although pseudotime approximates the ordering of cellular states, it lacks explicit directionality [25, 107]. A large number of these trajectory inference methods leverage only transcriptomic data. According to their underlying computational principles and the types of trajectories they aim to reconstruct, they can be broadly categorized into three classes: graph‐based methods, manifold learning methods, and principal curve methods.

Graph‐based methods construct networks to represent cell relationships, using similarity matrices or k‐nearest neighbor (KNN) graphs from single‐cell transcriptomic or epigenomic data. These graphs capture developmental trajectories, bifurcations, and lineage relationships [108, 109]. Monocle creates a minimum spanning tree (MST) through independent component analysis in reduced‐dimensional space, ordering cells along the longest path and defining branching events with marker genes or sample collection times [52]. Monocle 2, an upgraded version, uses reversed graph embedding for unsupervised learning of complex pseudotime trajectories [92]. Waterfall and TSCAN utilize cluster‐based MSTs for pseudo‐temporal cell ordering [93, 94]. Slingshot improves lineage inference and pseudotime estimation by combining stable techniques with multiple trajectory identification [47]. PAGA extends graph‐based approaches by linking clusters based on shared transcriptomic features, ideal for large datasets and early decision point analysis [95].

Manifold learning methods are powerful tools for cell lineage tracing, which assume that high‐dimensional gene expression data lie on a lower‐dimensional manifold. These techniques identify this manifold and infer trajectories by mapping cells onto it [108]. Recent methods such as MARGARET use deep unsupervised learning to reconstruct complex trajectory topologies and infer cell fate plasticity [96]. MIOFlow integrates dynamic models, manifold learning, and optimal transport to learn stochastic population dynamics from static snapshots [97]. Diffusion Pseudotime (DPT) applies diffusion maps and random walks to measure transitions between cells, reconstructing lineage branching and developmental progression [98]. Wishbone combines diffusion maps with KNN graphs to identify bifurcating trajectories and assign pseudotime values to cells [99]. These methods have improved the ability to reconstruct developmental trajectories and study cellular dynamics in contexts such as hematopoiesis, embryogenesis, and disease progression.

Principal curves, originally defined as self‐consistent smooth curves through the center of a data distribution, were later redefined to minimize the expected squared distance to random points [110]. These methods infer cell trajectories from scRNA‐seq data by fitting smooth curves, capturing the continuous progression of cellular states [103], but are limited to nonbranching lineages [47]. For example, Embeddr uses Laplacian eigenmaps with principal curves to create a low‐dimensional embedding that is robust to noise in single‐cell data [100]. Similarly, SCORPIUS integrates dimensionality reduction with principal curve fitting to infer linear developmental trajectories, effectively capturing continuous cellular progressions [101]. Extending these approaches, ElPiGraph employs elastic principal graphs to model complex, branching trajectories, accommodating the multifaceted nature of cellular differentiation [102]. Further, scShaper applies an ensemble approach to generate smooth pseudotime orderings, outperforming traditional principal curve methods on nonlinear trajectories and achieving higher accuracy in cell ordering and differential gene expression analysis [103].

In addition to scRNA‐seq‐based lineage tracing, natural genomic variations, such as single nucleotide variants (SNVs) and copy number variations (CNVs), serve as natural tags for reconstructing tumor lineages, differentiating them from experimental methods using reporter genes or barcode tracking [111, 112, 113, 114, 115]. SNP‐ and CNV‐based lineage tracing methods are essential for understanding tumor evolution, as clones exhibit different genetic compositions, and detecting clonal heterogeneity from high‐throughput data is crucial for cancer research [116, 117]. The EXPANDS method infers tumor evolution and subclonal diversity by estimating the proportion of cells with specific mutations, modeling cell frequencies as probability distributions. It identifies mutations that accumulate before clonal expansion [118]. The minimal event distance aneuploidy lineage tree (MEDALT) uses single‐cell copy number profiles to infer cell population evolution, incorporating lineage species formation analysis to identify adaptive changes [119]. MEDALT outperforms phylogenetic methods in copy number lineage reconstruction and predicts patient survival in triple‐negative breast cancer. Similarly, MEDICC2 utilizes haplotype‐specific somatic CNVs in single‐cell or bulk data to infer evolutionary trees and whole‐genome duplications [120]. MEDICC2 avoids the infinite‐site assumption, accommodates multiple mutations and parallel evolution, and proves accurate in simulating data from 2780 tumors. CONET is a probabilistic model that jointly infers copy number events and evolutionary trees [121]. Using efficient MCMC algorithms, CONET excels in tree reconstruction, breakpoint detection, and copy number detection, outperforming other methods in both simulated and experimental data.

#### 3.2.2 With RNA velocity

RNA velocity is a computational method used to infer the future state of a cell based on the analysis of splicing kinetics and gene expression dynamics [122, 123, 124]. By measuring both spliced and unspliced mRNA levels, RNA velocity predicts the direction and rate of cell state transitions, allowing for a more dynamic understanding of cell differentiation, reprogramming, and disease progression. Unlike traditional methods that rely on static gene expression profiles, RNA velocity provides a temporal dimension to scRNA‐seq data, enhancing the ability to model cellular trajectories in both development and disease.

One prominent method in RNA velocity analysis is velocyto, a tool that computes RNA velocity from scRNA‐seq data by modeling gene splicing kinetics [48]. It calculates the unspliced‐to‐spliced ratio for each gene to predict a cell’s future transcriptional state and trajectory. This method has been widely applied in studying developmental processes, such as neuronal differentiation, offering higher resolution in inferring cellular transitions. scVelo improves upon velocyto by integrating dynamic modeling and kinetic data, providing more robust RNA velocity estimates and enhanced trajectory resolution [104]. It is used in regenerative medicine and developmental studies, offering deeper insights into dynamic cellular processes.

Another key RNA velocity‐based method is dynverse, which integrates RNA velocity with dimensionality reduction techniques such as PCA, *t*‐SNE, and UMAP to visualize cell trajectories [25]. By combining RNA velocity with other trajectory inference methods, dynverse reconstructs complex cellular pathways, offering deeper insights into cell fate decisions. It has been applied to developmental biology, cancer research, and stem cell differentiation. Lange et al. introduced CellRank for single‐cell fate mapping, simulating cell state transitions while aligning with the global structure of the phenotype manifold (KNN graph based on gene expression similarity) [125]. CellRank combines RNA velocity with trajectory inference, accounting for the stochastic nature of cell fate decisions. In pancreatic development, it detects initial, intermediate, and terminal populations, predicts fate potential, and visualizes continuous gene expression trends across lineages.

RNA velocity can also be enhanced through experimental methods to improve the temporal resolution of the lineage tree. By combining RNA velocity with CRISPR‐induced mutations or barcodes, it is possible to provide information about a cell’s past lineage and predict its future differentiation paths. The integration of these two methods allows for tracking both the ancestral and future states of cells. For example, Wang et al. introduced PhyloVelo [126], a computational framework that estimates transcriptomic dynamics by using monotonically expressed genes (MEGs) or expression patterns of genes that increase or decrease over phylogenetic time without cycling. By integrating scRNA‐seq data with lineage information, PhyloVelo identifies MEGs and reconstructs the transcriptomic velocity field. When applied to seven lineage tracing datasets generated by CRISPR‐Cas9 editing, lentiviral barcoding, or immune repertoire analysis, PhyloVelo demonstrated high accuracy and robustness in inferring complex lineage trajectories, outperforming RNA velocity.

RNA velocity‐based methods fundamentally enhance computational approaches for cell lineage tracing by leveraging RNA velocity. Moreover, additional data from various sources and modalities, including spatial transcriptomics, can also be utilized for cell lineage tracing. For example, Karras et al. provided spatial and temporal maps of melanoma cell state diversity and trajectory, and by combining mouse genetics, single‐cell and spatial transcriptomics, lineage tracing, and quantitative modeling, it offers evidence for a tumor growth stratification model that reflects the cellular and mole‐cular logic behind the fate specification and differentiation of embryonic neural crest cells [127]. This study also decoded the complexity and heterogeneity of cell growth at attachment sites post‐birth using single‐cell transcriptomics and spatial datasets, revealing the molecular dynamics during the differentiation process of fibrocartilage. With the current spatial transcriptomics data, these findings offer a transcriptional resource that will support future mechanistic studies on intercalary deve‐lopment and may help identify strategies to enhance rotator cuff healing outcomes.

Despite its advantages, RNA velocity has certain limitations. One of the main challenges is the need for high‐quality data to accurately measure both spliced and unspliced transcripts. In addition, interpreting RNA velocity predictions requires careful consideration of factors such as gene expression noise, which can affect the accuracy of predictions in more heterogeneous populations of cells. Furthermore, RNA velocity methods currently rely heavily on the assumption that splicing rates are relatively constant, which may not always hold true under all biological conditions. Nevertheless, RNA velocity has become a powerful tool for studying the dynamics of cellular transitions and is expected to continue playing a major role in understanding deve‐lopmental processes, cellular reprogramming, and disease mechanisms, particularly when combined with other high‐dimensional single‐cell technologies [25, 48, 104].

## 4 APPLICATIONS AND INTERDISCIPLINARY INSIGHTS

### 4.1 Applications

Cell lineage tracing has become an indispensable tool in various fields of biology and medicine, enabling researchers to unravel the complex processes of deve‐lopment, regeneration, and disease progression.

One of the primary applications is in developmental biology, where lineage tracing provides a detailed understanding of how tissues and organs form during embryogenesis [3, 128]. For instance, long‐term lineage tracing has shown that the melanocyte stem cell (McSC) system is maintained by recovered McSCs and not by retained stem cells that are essentially unaffected by reversible changes [129]. During aging, there is an accumulation of chain‐like McSCs, and these McSCs do not contribute to the regeneration of melanocyte progeny. These results identify a novel model in which dedifferentiation is integral to homeostatic stem cell maintenance and suggest that modulation of McSC activity may represent a novel approach to prevent hair graying. Kasmani et al. investigated the multifaceted process of CD8^+^ T cell differentiation, in which progenitor CD8^+^ T cells maintained end‐effector and exhausted CD8^+^ T cell subsets by bifurcating into intermediate phenotypes [130]. This may have represented functional adaptations that occurred when antigen presentation and inflammatory signals persisted, such as during chronic infections and cancer.

In regenerative medicine, lineage tracing has played a pivotal role in identifying stem cell niches and tracking the dynamics of tissue repair and regeneration [12, 131, 132, 133]. For example, long‐term lineage tracing studies in mouse models elucidated the contributions of stem cells to tissue homeostasis and regeneration following injury [3, 12]. These findings had direct implications for the development of cell‐based therapies, highlighting the potential of targeting specific stem cell populations to enhance regenerative capacity. Understanding the underlying mechanisms and long‐term consequences of adaptive and maladaptive kidney repair after an abrupt decrease in kidney function was critical to kidney health. Gerhardt et al. used lineage tracing and mononucleotide multiomics to analyze genome organization and gene activity at single‐cell resolution, providing new insights into the regulation of kidney injury repair [134]. Lotto et al. noted that lineage tracing effectively elucidated the hepatocyte lineage hierarchy, offering insights into the pathogenesis of liver disease and cancer, where developmental processes were involved in disease emergence and regeneration [135]. Future work would focus on translating this knowledge into optimized in vitro models of liver development and fine‐tuning regenerative medicine strategies to treat liver disease.

Oncology represents another critical area where lineage tracing has made significant contributions. Tumor lineage tracing using CRISPR barcoding has revealed the heterogeneity of cancer cell populations and the evolutionary dynamics of metastasis [14, 15, 136, 137]. These studies have provided valuable insights into how genetic and epigenetic changes drive tumor progression and resistance to therapy [16]. Moreover, lineage tracing combined with scRNA‐seq has been used to identify subpopulations of cancer cells with distinct metastatic potential, aiding in the development of more targeted therapeutic strategies [13, 17]. For example, gene expression networks were created from hundreds of mouse tissue samples (both normal and tumor) and combined with lineage tracing and scRNA‐seq to determine cellular state convergence in precancerous tumor cells expressing markers of lineage plasticity and drug resistance [138]. Zhou et al. combined in vivo lineage tracing and scRNA‐seq to reveal the heterogeneity and dynamics of Prom1^+^ HCC cells, providing insights into the mechanistic role of malignant liver cancer stem cell (CSC)‐like cells in hepatocellular carcinoma (HCC) progression [139]. By integrating multiple scRNA‐seq studies and performing robust lineage tracing assays, Huang et al. identified antigen‐presenting cancer‐associated fibroblasts derived from mesothelial cells [140].

Tumor clonal tracking is crucial for understanding tumor evolution, clonal dynamics, and resistance [14, 15, 141]. Gutierrez et al. developed ClonMapper, which combines clonal characterization and separation to profile thousands of clones in heterogeneous populations [142]. This system identified subpopulations in chronic lymphocytic leukemia (CLL) with distinct clonal compositions and chemotherapy survival trajectories, patterns also seen in primary human cases. Werdyani et al. analyzed whole‐genome INDEL/CNV profiles in colorectal cancer patients and found that INDEL and CNV polymorphisms influenced cancer susceptibility and prognosis, highlighting new variants related to relapse risk [143]. Zucker et al. used SNV data and copy number estimates to detect clonal heterogeneity [144]. Understanding the clinical significance of clonal heterogeneity, especially in cancers such as CLL, is key for identifying resistance and progression mechanisms. Tumors with multiple clones can drive disease progression or acquire resistance mutations over time. Miller et al. focused on copy‐neutral LOH regions to quantify VAFs and infer clonality in acute myeloid leukemia (AML) and breast cancer [145]. This approach detected subclones not evident in single primary tumor samples but present at disease onset. Kester et al. integrated single‐cell WGS with viral lineage tracking to analyze CNV, SNV, and viral lineage barcodes in 1641 cells, constructing a high‐resolution clonal evolutionary tree [146]. This method identified key chromosomal aberrations and recurrent mutations in colon cancer organoid models, offering insights into tumor evolution and potential clinical targets.

Cell lineage tracing has emerged as a versatile and transformative tool, with applications ranging from fundamental biology to translational medicine. As experimental and computational techniques continue to evolve, its impact on understanding cellular dynamics and its potential to inform therapeutic strategies will keep growing.

### 4.2 Interdisciplinary insights

Cell lineage tracing is inherently intertwined with various disciplines in modern biology and biomedical research. Its integration with single‐cell omics, epigenetic profiling, and disease modeling highlights its pivotal role in advancing the understanding of cellular dynamics, differentiation, and disease progression.

One key relationship is with single‐cell omics technologies, such as scRNA‐seq, single‐cell epigenomics, and single‐cell proteomics. These methods, when combined with lineage tracing, provide a multidimensional view of cellular states and transitions. For example, integrating lineage information with transcriptomics allows researchers to identify lineage‐specific gene expression patterns and reconstruct cell fate decisions with greater resolution [35, 48]. Epigenomic profiling, on the other hand, helps elucidate how chromatin states influence lineage hierarchies and cellular plasticity [36, 64]. This integration is crucial for understanding developmental processes and how cells respond to environmental cues, such as stress or injury [13].

In disease modeling, lineage tracing has become a transformative tool [147, 148, 149, 150]. It allows researchers to trace the origins and evolution of pathological conditions, such as cancer metastasis or tissue degeneration, providing critical insights into disease mechanisms [3, 14]. For example, tumor lineage tracing using CRISPR‐Cas9 barcodes has revealed how genetic heterogeneity contributes to drug resistance and relapse in cancer therapy [15, 16]. Similarly, in regenerative medicine, lineage tracing has been used to identify stem cell niches and track the dynamics of cellular regeneration following injury or transplantation [12, 36].

Another critical intersection is with computational biology and bioinformatics. The analysis of lineage tracing data requires sophisticated algorithms to process large‐scale single‐cell datasets, often integrating RNA velocity or dimensionality reduction techniques [48, 104]. Tools such as PAGA95 and Slingshot [47] were developed to infer lineage trajectories while accounting for branching events and non‐linear transitions. These computational advancements enable researchers to not only reconstruct lineage trees but also predict future cell states, bridging the gap between static and dynamic cellular studies.

Furthermore, lineage tracing contributes to the study of epigenetic inheritance and its role in cell fate decisions. Techniques such as EpiTrace [64] leverage CRISPR‐Cas9 to simultaneously trace lineage and track epigenetic modifications, uncovering the interplay between genetic and epigenetic factors in shaping cellular identities. This integration is particularly valuable in understanding transgenerational inheritance and the long‐term impacts of environmental factors on cell fate [13, 27].

Finally, cell lineage tracing addresses broader biological questions about cellular heterogeneity and evolution. By mapping lineage relationships across diverse tissues and organisms, it provides a framework for comparing developmental processes and identifying conserved mechanisms of cellular differentiation [25, 35]. This comparative approach is instrumental in bridging developmental biology and evolutionary studies, enabling researchers to explore how cellular lineages adapt across different species and environmental contexts [3, 4].

In summary, the interdisciplinary applications of cell lineage tracing emphasize its transformative potential in modern biology. By integrating lineage information with multi‐omics, computational methods, and disease modeling, it provides unparalleled insights into cellular dynamics, offering a foundation for breakthroughs in developmental biology, regenerative medicine, and oncology.

## 5 CHALLENGES

While cell lineage tracing has made remarkable progress in understanding cellular dynamics, several critical challenges remain that hinder its broader application and impact.

Firstly, the accuracy of lineage reconstruction remains a significant hurdle. Despite advances in ex‐perimental methods, such as CRISPR‐Cas9 barcoding and RNA velocity, the inherent noise in single‐cell data often leads to ambiguity in lineage assignments. Overlapping or saturated barcodes, as well as technical artifacts, can obscure true lineage relationships, making it difficult to achieve high‐resolution lineage trees [35, 36].

Building on this, a second major challenge lies in the temporal resolution of lineage tracing. Capturing the dynamic processes of cell state transitions requires tools that can resolve temporal changes with precision. While RNA velocity methods have introduced a dynamic dimension to lineage tracing, their reliance on assumptions about splicing kinetics and steady‐state conditions limits their applicability in heterogeneous or rapidly evolving systems [48]. Temporal resolution is critical for understanding developmental processes and for predicting future cell states, highlighting the need for more robust methods to address this gap.

Thirdly, the integration of lineage data with multi‐omics approaches poses a substantial challenge [151]. Combining transcriptomic, epigenomic, and proteomic data with lineage information provides a multidimensional view of cellular states, yet the computational and experimental complexities involved are significant. Data integration often requires specialized algorithms and large‐scale validation to ensure fidelity, particularly when dealing with diverse biological systems [13, 64]. This limits the accessibility of such approaches to broader research communities.

Lastly, scalability remains an overarching challenge for lineage tracing methods. As the size and complexity of datasets increase, methods must adapt to analyze entire tissues, organs, or even whole organisms without sacrificing resolution or accuracy. Current tools often struggle to scale effectively, leading to data saturation or computational bottlenecks in large‐scale studies [34]. Addressing this challenge is critical for extending the applicability of lineage tracing to more complex bio‐logical systems.

In summary, the challenges of accuracy, temporal resolution, multi‐omics integration, and scalability are deeply interrelated. Overcoming these obstacles requires continued innovation in both experimental techniques and computational frameworks, which will be key to unlocking the full potential of cell lineage tracing in developmental biology, regenerative medicine, and disease modeling.

## 6 DISCUSSION

In this paper, we review both experimental and computational cell lineage tracing methods. The experimental methods encompass traditional and modern techniques used to label and track cells within biological systems, such as fluorescent protein reporters, Cre‐loxP recombination systems, and CRISPR‐Cas9‐based barcoding. The computational methods involve bioinformatic tools and algorithms designed to analyze complex datasets generated from high‐throughput experiments and to reconstruct lineage relationships through computational modeling.

While significant advancements were made in both experimental and computational lineage tracing, several limitations persist that hinder a comprehensive understanding of cellular development and differentiation. One major limitation of current experimental methods is the trade‐off between resolution and scalability [55]. Techniques that offer single‐cell resolution often lack the capacity to track large populations over extended periods, whereas methods suitable for large‐scale studies may not provide detailed insights into individual cell fates [27]. Additionally, some labeling techniques may introduce perturbations that alter normal cellular behavior, leading to artifacts in lineage reconstruction.

Computational methods, on the other hand, face challenges related to data complexity and integration. The high dimensionality of omics datasets, such as transcriptomics, epigenomics, and proteomics, requires sophisticated algorithms capable of handling noise and variability inherent in biological systems. Reconstructing accurate lineage trees from scRNA‐seq data is complicated by factors such as transcriptional heterogeneity and temporal dynamics [152]. Moreover, most computational approaches rely on assumptions that may not hold true across different cell types or experimental conditions, potentially leading to biased or incomplete lineage models.

To address these limitations, future improvements should focus on enhancing the resolution, accuracy, and scalability of both experimental and computational techniques. One promising direction is the integration of transcriptomic data with CRISPR‐Cas9‐based lineage tracing. Combining single‐cell transcriptomics with genetic barcoding allows for the simultaneous capture of a cell’s lineage history and its gene expression profile [153, 154]. This dual information can provide a more comprehensive understanding of how genetic and epigenetic factors influence cell fate decisions. Advances in multiplexed imaging and spatial transcriptomics [155, 156] also offer the potential to map lineage relationships within the spatial context of tissues, adding another layer of information to lineage studies.

Another area requiring attention is the development of non‐invasive and minimally perturbative labeling techniques. Innovations in synthetic biology and chemical biology could yield new tools for cell labeling that do not interfere with normal cellular functions [157, 158]. For computational methods, the implementation of machine learning and artificial intelligence algorithms can improve the analysis of complex datasets [159, 160, 161]. Deep learning approaches [162, 163], for instance, have the potential to uncover hidden patterns in high‐dimensional data that traditional methods might miss.

Interdisciplinary collaboration between experimentalists and computational scientists is essential to propel the field forward. Developing standardized frameworks and data sharing platforms can facilitate the comparison and validation of lineage tracing studies across different systems and laboratories. Additionally, ethical considerations regarding genetic manipulation and data privacy must be addressed, especially as techniques become more powerful and accessible [164, 165].

In conclusion, while current cell lineage tracing methods have significantly advanced the understanding of biological development and disease, there is ample room for improvement. By addressing the limitations of existing technologies and embracing innovative approaches that integrate multiple data types, researchers can achieve a more subtle and holistic view of cellular dynamics. This progress will not only enhance basic scientific knowledge but also translate into clinical applications in regenerative medicine, oncology, and personalized therapy, ultimately benefiting human health and disease management.

## AUTHOR CONTRIBUTIONS

**Shanjun Mao**: Formal analysis; investigation; writing—original draft. **Chenyang Zhang**: Formal analysis; investigation. **Runjiu Chen**: Formal analysis; investigation. **Shan Tang**: Formal analysis; investigation. **Xiaodan Fan**: Conceptualization; supervision; writing—review and editing. **Jie Hu**: Methodology; supervision; writing—review and editing.

## CONFLICT OF INTEREST STATEMENT

The authors declare no conflicts of interest.

## ETHICS STATEMENT

This review article does not involve any research related to human or animal subjects.

## Data Availability

Data sharing is not applicable to this article as no new data were created or analyzed in this study.
